# Editorial for the Special Issue on Micro/Nanofluidic and Lab-on-a-Chip Devices for Biomedical Applications

**DOI:** 10.3390/mi13101718

**Published:** 2022-10-12

**Authors:** Violeta Meneses Carvalho, Senhorinha Teixeira, João E. Ribeiro

**Affiliations:** 1 MEtRICs, Mechanical Engineering Department, Campus de Azurém, University of Minho, 4800-058 Guimarães, Portugal; 2 ALGORITMI Research Centre/LASI, Department of Production Systems, University of Minho, 4800-058 Guimarães, Portugal; 3 Microelectromechanical Systems Research Unit (CMEMS-UMinho), School of Engineering, Campus de Azurém, University of Minho, 4800-058 Guimarães, Portugal; 4 Campus de Santa Apolónia, Instituto Politécnico de Bragança, 5300-253 Bragança, Portugal; 5 Centro de Investigação de Montanha (CIMO), Campus de Santa Apolónia, Instituto Politécnico de Bragança, 5300-253 Bragança, Portugal; 6 Laboratório Associado Para a Sustentabilidade e Tecnologia em Regiões de Montanha (SusTEC), Campus de Santa Apolónia, Instituto Politécnico de Bragança, 5300-253 Bragança, Portugal

Micro/Nanofluidic and lab-on-a-chip devices have been increasingly used in biomedical research [1]. Because of their adaptability, feasibility, and cost-efficiency, these devices can revolutionize the future of preclinical technologies. Furthermore, they allow insights into the performance and toxic effects of responsive drug delivery nanocarriers to be obtained, which consequently allow the shortcomings of two/three-dimensional static cultures and animal testing to be overcome and help to reduce drug development costs and time [2,3,4]. With the constant advancements in biomedical technology, the development of enhanced microfluidic devices has accelerated, and numerous models have been reported.

Given the multidisciplinary of this Special Issue (SI), papers on different subjects were published making a total of 14 contributions, 10 original research papers, and 4 review papers. The review paper of Ko et al. [1] provides a comprehensive overview of the significant advancements in engineered organ-on-a-chip research in a general way while in the review presented by Kanabekova and colleagues [2], a thorough analysis of microphysiological platforms used for modeling liver diseases can be found. To get a summary of the numerical models of microfluidic organ-on-a-chip devices developed in recent years, the review presented by Carvalho et al. [5] can be read. On the other hand, Maia et al. [6] report a systematic review of the diagnosis methods developed for COVID-19, providing an overview of the advancements made since the start of the pandemic.

In the following, a brief summary of the research papers published in this SI will be presented, with organs-on-a-chip, microfluidic devices for detection, and device optimization having been identified as the main topics. 

Some researchers focused on the development of advanced microfluidic devices which are devised to reconstruct the tissue architecture of organs biochemically and biophysically. For instance, Kuriu and co-workers [4] worked on the development of a microfluidic device to mimic the small intestine tract with villi to obtain insights into fluid flow by using particle image velocimetry. Through these experiments, it was possible to verify that microbeads tend to stick to the side surface of the villi, which can explain the relationship between fluid flow and the settlement of gut bacteria on the villi. Komen and colleagues [7], on the other hand, established an alternative to cancer xenografts due to ethical considerations and a lack of accuracy to predict physiological responses. The authors created a microfluidic device integrated with a U-shaped well for having a single spheroid and exposed it to a dynamic environment and to an in vivo-like concentration of oxaliplatin, a medication commonly used to treat colorectal cancer, and compared it to an in-vivo cancer xenograft. The effect of oxaliplatin on growth inhibition, proliferation, and apoptosis markers was evaluated. In terms of growth inhibition, the on-chip results were comparable to xenograft studies. Regarding the proliferation and apoptosis markers, a similar response was also observed which proved the potential of microfluidic devices to reduce the use of cancer xenografts for cancer research. Meanwhile, Callegari and colleagues [8] investigated the electrophysiological activity of neuronal populations to electrical stimulation. For this purpose, cortical and hippocampal neurons were established to reconstruct interconnected sub-populations. The results showed that cortical assemblies were more reactive than hippocampal ones. Through these results, the authors showed that the results depend on neuronal structure when electrical stimulation experiments are conducted. Despite the previous works presenting interesting results, the fabrication process of advanced microfluidic devices has constantly evolved, and the use of 3D (bio)printing has increased over the years. Lutsch et al. [9] presented an alternative to the regularly used PDMS casting method. The authors investigated different resins by conducting cytotoxicity, cytocompatibility, and HET CAM assay, but poly-(ethylene glycol)-diacrylate (PEGDA) stood out. This material provided excellent results and allowed for the acceleration and improvement of the current fabrication processes. 

Other authors have focused on the use of microfluidic devices for detection purposes. Li et al. [10] designed an ease-of-use portable microfluidic device that combines microarray and microfluidics for point-of-care detection of several protein biomarkers in serum samples. The results were promising and showed similar outputs to those measured by the commercial method used at the clinic. Similarly, Sitkov [11] constructed a microfluidic biosensor system with peptide aptamers for protein biomarker detection of diseases in biological fluids. Through in silico techniques, the authors simulated the peptide aptamer for troponin T and its non-fluorescent digital twin and tested the device design. Since the simulation results were promising, a laboratory sample of the biosensor was designed and manufactured, but further experiments are needed. In a similar line of research, Zimmers and co-workers [12] conceived a novel diagnostic platform for the detection of target DNA. In brief, the target DNA is captured by magnetic beads and then loaded into a microfluidic reaction tape with the other reaction solutions. The device was able to detect 5 fM target DNA as well as *Schistosoma mansoni* DNA. Despite additional tests being needed, this technique requires less hands-on time and does not need an additional control reaction like current methods do. A different detection system was proposed by Saha and co-workers [13], but in this case for lactate quantification using a colorimetric assay. This can be used for evaluating the welfare of muscles and oxidative stress levels. The authors developed a wearable patch due to the relationship between sweat and blood lactate levels. For instance, during rest, the osmotic disc (hydrogel) can extract fluid from the skin via osmosis and deliver it to the paper, while during exercise, the paper can collect sweat even in the absence of the hydrogel patch. It was found that the molar concentration of lactate in sweat is correlated to sweat rate and that the measurements are more viable during high-intensity exercise. This constitutes an interesting technology that can be used for athlete monitoring.

The optimization of microfluidic devices has also been explored by some authors. Grigorev et al. [3] investigated how to achieve adequate flow rates for trapping single cells in a microfluidic chip. The authors applied a generative design methodology with an evolutionary algorithm and validated the device with experimental data. The experiments proved the efficiency of the device with 4 out of 4 RBCs trapped. On the other hand, Tsai and colleagues [14] focused on microfluidic devices for bacterial growth and how to guarantee microscope stability for long-term imaging of bacterial dynamics. For this purpose, an optimized integrated multi-level microfluidic chip was developed. The authors used a stabler microscopy immersion oil, and images were captured with a focally stable time-lapse for 72 h. 

To conclude, we would like to acknowledge all the authors for their contribution to the success of this SI as well as the reviewers whose feedback helped to improve the quality of the published papers. We would also like to recognize Ms. Min Su from the Micromachines publishing office for her endless assistance and help in disseminating this SI.
